# Cognitive Motor Dissociation in Disorders of Consciousness

**DOI:** 10.1056/NEJMoa2400645

**Published:** 2024-08-15

**Authors:** Yelena G Bodien, Judith Allanson, Paolo Cardone, Arthur Bonhomme, Jerina Carmona, Camille Chatelle, Srivas Chennu, Mary Conte, Stanislas Dehaene, Paola Finoia, Gregory Heinonen, Jennifer Hersh, Evelyn Kamau, Phoebe Lawrence, Victoria C. Lupson, Anogue Meydan, Benjamin Rohaut, William R Sanders, Jacobo D Sitt, Andrea Soddu, Mélanie Valente, Angela Velazquez, Henning U Voss, Athina Vrosgou, Jan Claassen, Brian L Edlow, Joseph J Fins, Olivia Gosseries, Steven Laureys, David Menon, Lionel Naccache, Adrian M Owen, John Pickard, Emmanuel A Stamatakis, Aurore Thibaut, Jonathan D Victor, Joseph T Giacino, Emilia Bagiella, Nicholas D Schiff

**Affiliations:** 1 Department of Physical Medicine and Rehabilitation, Spaulding Rehabilitation Hospital, Charlestown MA, USA; 2 Center for Neurotechnology and Neurorecovery, Massachusetts General Hospital, Boston MA, USA; 3 Department of Physical Medicine and Rehabilitation, Harvard Medical School, Boston MA, USA; 4 Department of Neurology, Massachusetts General Hospital and Harvard Medical School, Boston MA, USA; 5 Department of Neurosciences, Addenbrookes Hospital, Cambridge UK; 6 Department of Clinical Neurosciences, University of Cambridge, UK; 7 Coma Science Group, GIGA Consciousness Research Unit, University and University Hospital of Liège, Liège, Belgium; 8 Centre du Cerveau, University Hospital of Liège, Liège, Belgium; 9 Department of Neurology, Columbia University Irving Medical Center, Presbyterian Hospital, New York, NY, USA; 10 European Research Council Executive Agency, Brussels, Belgium*; 11 Division of Neurosurgery, School of Clinical Medicine, University of Cambridge, Cambridge, UK; 12 School of Computing, University of Kent, Kent, UK; 13 Feil Family Brain and Mind Research Institute, Weill Cornell Medicine, New York, NY, USA; 14 Collège de France, Université Paris-Sciences-Lettres, Paris, France; 15 Division of Medical Ethics, Weill Cornell Medicine, New York, NY, USA; 16 Wolfson Brain Imaging Centre, University of Cambridge, Cambridge, UK; 17 Sorbonne Université, Institut du Cerveau—Paris Brain Institute—ICM, INSERM, CNRS, Paris, France; 18 AP-HP, Hôpital Pitié-Salpêtrière, DMU Neurosciences, Paris, France; 19 Physics & Astronomy Department, University of Western Ontario, London ON, Canada; 20 Western Institute for Neuroscience, University of Western Ontario, London ON, Canada; 21 Department of Radiology, Weill Cornell Medicine, New York, NY, USA; 22 Athinoula A. Martinos Center for Biomedical Imaging, Massachusetts General Hospital, Charlestown, MA, USA; 23 The Rockefeller University Hospital, New York, NY, USA; 24 Yale Law School, New Haven, CT; 25 CERVO Brain Research Centre 2601, de la Canardière, Québec Canada G1J 2G3; 26 Consciousness Science Institute, Hangzhou Normal University, Hangzhou, Zhejiang, China; 27 Division of Anaesthesia, Department of Medicine, University of Cambridge, Cambridge, UK; 28 Department of Neurology, Weill Cornell Medicine, New York, NY, USA; 29 Department of Population Health Science & Policy, Center for Biostatistics, Icahn School of Medicine at Mount Sinai, New York, NY, USA

## Abstract

**Background:**

Patients with brain injury who are unresponsive to command may perform cognitive tasks that are detected by functional magnetic resonance imaging (fMRI) and electroencephalography (EEG). This phenomenon, known as cognitive motor dissociation, has not been systematically studied in a large cohort of patients with disorders of consciousness.

**Methods:**

In this prospective cohort study conducted at six international centers, we collected clinical, behavioral, and task-based fMRI and EEG data on a convenience sample of 353 adults with disorders of consciousness. Sixty-six percent of participants had only fMRI or EEG and 34% had both. We determined the proportion of participants with and without observable responses to verbal commands who had responses to command on task-based fMRI or EEG.

**Results:**

Participants’ median age was 37.9 years, median time from injury was 7.9 months (26% within 28 days of injury), and 50% had a traumatic etiology. Of 241 participants without observable response to commands (i.e., behavioral diagnosis of coma, vegetative state, or minimally conscious state minus), we detected cognitive motor dissociation in 60 (25%; n=11 assessed with fMRI only, n=13 with EEG, and n=36 with both methods). Cognitive motor dissociation was associated with younger age, longer chronicity, and traumatic etiology. In contrast, of 112 participants with observable response to commands, task-based fMRI or EEG responses were present in 43 (38%).

**Conclusions:**

Approximately one in four participants without observable response to commands performed a cognitive task on fMRI or EEG, compared with one in three participants with observable response to commands.

## Introduction

Cognitive motor dissociation^1^ is an established phenomenon^2–4^ that describes individuals with severe brain injury who are observed to be behaviorally unresponsive to commands, yet demonstrate brain activation on functional magnetic resonance imaging (fMRI) or electroencephalography (EEG) when presented with cognitive tasks, such as motor imagery commands. Failing to identify cognitive motor dissociation in patients with disorders of consciousness could affect decisions related to withdrawing life-sustaining treatment, goals of care, and clinical management. Evidence of cognitive motor dissociation may prompt more thorough investigation of subtle behaviors that are under volitional control,^5^ uncovering potential avenues for communication and patient autonomy.

In prior studies, cognitive motor dissociation was observed in 10-20% of persons with a disorder of consciousness,^3,6–9^ a finding demonstrated in both the acute^10,11^ and chronic^12^ stages of recovery as well as across etiologies.^9^ Detection of cognitive motor dissociation has been associated with more rapid recovery and better outcome at 1-year post-injury.^11,13^ To be detected on fMRI or EEG, responses to command must be sustained and require not only language comprehension but likely more cognitive processing (e.g., short-term memory, attention, persistence; Supplementary Appendix Table S1) than responding to a single command at the bedside. Identifying that a patient who otherwise appears unconscious has the capacity for cognitive processing may mitigate emotional suffering when their clinical team and family recognizes that they are aware and treat them as such. The harm in assuming an unresponsive patient is also unaware has been previously described.^14^ Recent international clinical guidelines vary in their level of endorsement of fMRI and EEG for detecting cognitive motor dissociation, from supporting their use^15,16^ to proposing that these techniques should be further studied prior to their application to routine medical practice.^17^

Most prior cognitive motor dissociation studies were conducted at a single site with relatively small cohorts.^3,6,7,9–11,18,19^ Our consortium determined the proportion of cognitive motor dissociation in a multi-center, multi-national cohort of participants with disorders of consciousness who were assessed at specialized centers that have the capability of studying this phenomenon.

## Methods

### Sites and Participants

Six multi-national sites contributed behavioral and task-based fMRI and/or EEG data to a centrally-curated database from 2006 to 2023. Participants were adults (≥18 years) with a disorder of consciousness recruited from intensive care units, hospital wards, rehabilitation facilities, nursing homes, and the community. Exclusion criteria at all sites were: 1) prior neurological or psychiatric disease, and 2) contraindication for MRI/EEG (as appropriate based on modalities used at each site; e.g. for fMRI, inability to lay flat or ferrous metal implants). References for inclusion and exclusion criteria for each site are available in Supplementary Appendix Table S2 and criteria are further detailed in Supplementary Appendix Table S3. Sites received approval from local ethics review boards and followed local regulations to obtain surrogate consent for study participation. Participants may have been included in prior studies aimed at testing specific methodologies or answering different research questions (Supplementary Appendix Figure S1).

NS (Administrative Principal Investigator), AO, and SL planned the initial phase of the study in 2008; NS, AO, SL, LN, JC, ES, OG, BE, JA, JP, and JG were site Principal Investigators from 2011 to 2023. The REDCap multi-center database and analyses were designed by NS, EB, YB, JG, JA, ES, LN, JC, JP, DM, and OG. EB carried out the data analyses and had no role in data collection. All other authors participated in data acquisition and supported local site infrastructure. YB and NS wrote the first draft of the paper, which was further developed in discussion with JA, PC, JC, OG, DM, LN, JP, ES, JG, and EB. All authors reviewed the paper.

### Procedures

Trained study staff conducted behavioral assessments by administering the Coma Recovery Scale-Revised (CRS-R, Supplementary Appendix Table S4),^20,21^ a standardized measure with high interrater and test-retest reliability^20^ that is validated in multiple languages.^22,23^ The CRS-R is the preferred measure for assessment of level of consciousness across international guidelines, and was the means by which we assigned patients a disorders of consciousness diagnosis.^15–17^ CRS-R examiners were blind to fMRI and EEG assessment results.

Each of the six sites has experience designing fMRI and EEG studies for patients with disorders of consciousness and followed local, previously published, validated procedures for acquiring, analyzing, and interpreting these data (Supplementary Appendix Table S2). fMRI and EEG data processing and interpretation procedures were automated to minimize bias associated with subjective discrimination of positive from negative responses. fMRI data used established statistical cut-points and cluster-correction for multiple comparisons to reduce the potential for spurious activations to appear in the *a priori* established regions of interest. EEG analysis utilized either a comparison of power spectral density at each channel (corrected for multiple comparisons) or a machine learning algorithm. Trained study staff who were masked to the participants’ behavioral assessment conducted EEG artifact rejection. Prior to evaluating participants with disorders of consciousness, sites tested the fMRI^3,8,10^ and EEG^10,11,24,25^ acquisition and analytic methods in healthy participants to ensure positive responses were obtained in individuals with intact cognitive processing; across these studies, which included 5 - 16 healthy participants, 70-100% demonstrated responses to command on task-based fMRI or EEG.

We included participants who had: 1) at least one CRS-R score *and* 2) assessment of command-following via task-based fMRI and/or EEG (e.g., “imagine playing tennis”, “imagine opening and closing your hand”, “open and close your hand”, or visual/auditory discrimination; see Supplementary Appendix Table S2 and Table S3 for complete task-based query) within seven days of the CRS-R. If participants were tested across multiple days with either fMRI or EEG, we included only the best performance on the first day in our analyses. We also documented the number of participants for whom it was not possible to analyze or interpret any fMRI or EEG sessions (e.g., due to motion artifact). Study staff from each site entered data into a central REDCap (Research Electronic Data Capture)^26^ database housed at Icahn School of Medicine at Mount Sinai, the Data Coordinating Center. REDCap variables included: demographic and clinical characteristics, CRS-R subscale (auditory, visual, motor, oromotor/verbal, communication, and arousal) and total scores (Supplementary Appendix Table S4), number of task-based fMRI and/or EEG sessions attempted, and number of task-based fMRI and/or EEG sessions with a positive or negative result.

### Analysis

We divided participants into two groups based on whether or not responses to verbal commands or intelligible speech was observed on the CRS-R examination. Cognitive motor dissociation was operationally-defined as the absence of command-following and intelligible speech on the CRS-R (i.e., auditory subscale score <3, *and* visual subscale <5, *and* oromotor/verbal subscale <3, *and* communication subscale <1; Supplementary Appendix Table S4) in the setting of a positive response to at least one task-based fMRI *and*/*or* EEG paradigm.^1^ Applying this definition, only participants with a CRS-R diagnosis of coma, vegetative state (also referred to as unresponsive wakefulness syndrome), or minimally conscious state minus (i.e., participants with signs of conscious awareness, such as visual pursuit, but without responses to commands or intelligible verbal output, Supplementary Appendix Table S4)^27^ can be classified as having cognitive motor dissociation. We combined the diagnostic categories of coma and vegetative state as both indicate an unconscious state. We also evaluated task-based fMRI and EEG responses in participants with observable response to commands (a behavioral diagnosis of minimally conscious state plus [participants with signs of conscious awareness that include following commands or intelligible verbal output] or emerged from minimally conscious state [participants who use common objects in a functional manner or correctly respond to basic yes/no situational orientation questions]).

The preservation or recovery of multiple complex cognitive functions required to perform the fMRI/EEG tasks over minutes of sustained engagement (Supplementary Appendix Table S1) minimizes spurious responses on fMRI^28^ and EEG.^29,30^ This methodological approach results in a high rate of test failure (i.e., no fMRI or EEG response in patients with observable command-following, or healthy participants, i.e., “false negative”).^3,10,31,32^ Given this context, we interpret positive fMRI and EEG results in participants without observable response to commands (a behavioral diagnosis of coma, vegetative state, or minimally conscious state minus) as specific for cognitive motor dissociation, but at a potential cost of sensitivity.

We report descriptive characteristics of the sample and the proportion of all participants who demonstrated cognitive motor dissociation. We describe differences in cognitive motor dissociation rates by age, chronicity, CRS-R diagnosis, etiology, and site. We calculated kappa coefficients to determine the agreement between the behavioral diagnosis, task-based fMRI, and task-based EEG results. Confidence intervals are not adjusted for multiplicity and cannot be used in place of hypothesis testing.

## Results

**Figure 1:**
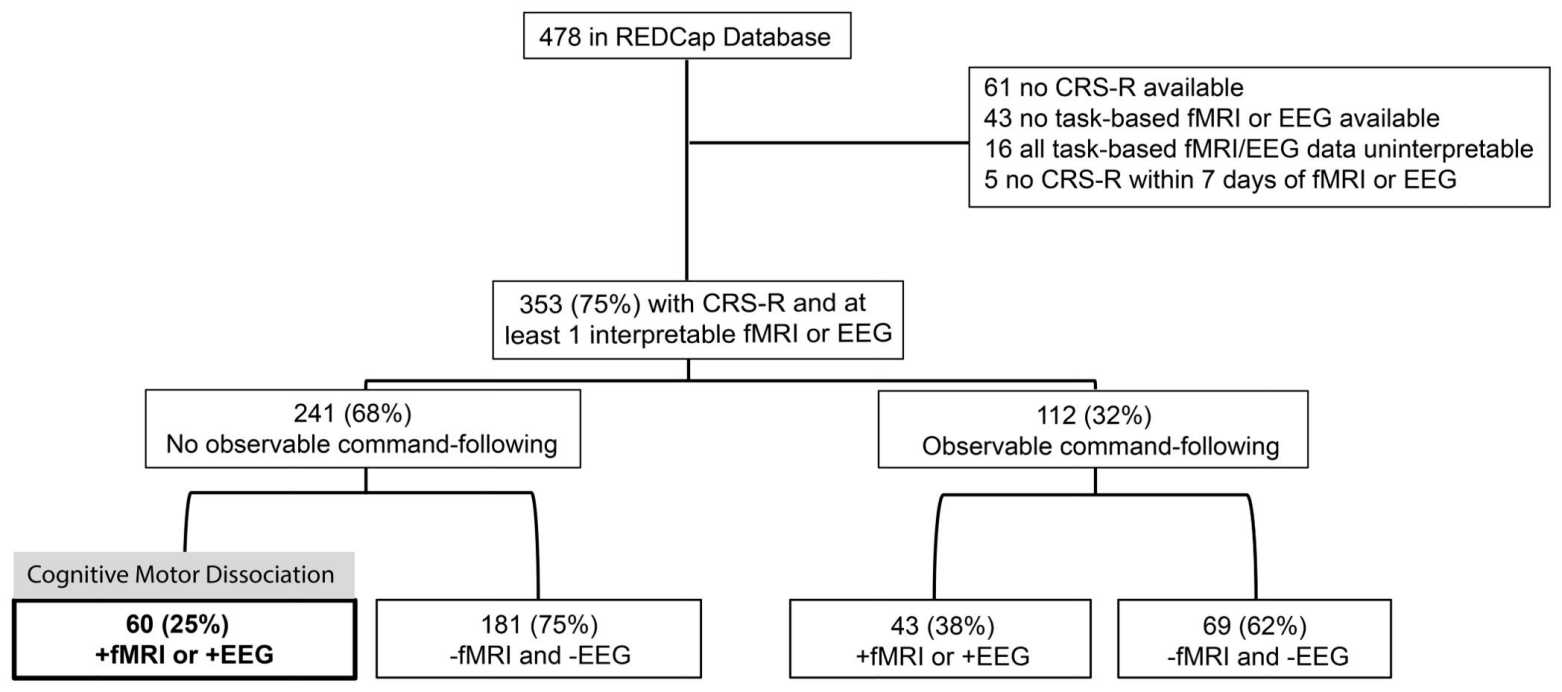
Participant Enrollment and Proportion with Cognitive Motor Dissociation Of 478 participants in the REDCap database, 353 were assessed with the CRS-R and with at least one command-following paradigm on fMRI or EEG within 7 days. Cognitive motor dissociation was observed in 25% of participants with no observable evidence of command-following (i.e., behavioral diagnosis of coma/vegetative state, [unconscious], or minimally conscious state minus [minimally conscious state without command-following], left branch). In participants with observable command-following (i.e., behavioral diagnosis of minimally conscious state plus [minimally conscious state with command-following] or emerged from minimally conscious state, right branch), a response to task-based fMRI or EEG was not detected in more than 60%. “+fMRI or +EEG” indicates that at least one assessment (either fMRI or EEG regardless of whether participants had one or both of these assessments) was positive. “-fMRI and -EEG” indicates that for participants with fMRI only, the fMRI assessment was negative; for participants with EEG only, the EEG assessment was negative; for participants with both fMRI and EEG, both assessments were negative. **Abbreviations**: CRS-R *Coma Recovery Scale-Revised*, EEG *electroencephalography*, fMRI *functional magnetic resonance imaging*

The central database included 478 participants of whom 125 were excluded from the current study (n=61 with no CRS-R score, n=43 with no task-based fMRI or EEG data, n=16 with uninterpretable fMRI and/or EEG data, and n=5 with a CRS-R score that was obtained more than 7 days before or after fMRI/EEG; Figure 1). Characteristics of included participants (n=353) are provided in Table 1 and Supplementary Appendix Figure S2. Supplementary Appendix Figure S1 describes the 232 (66%) participants who were included in prior studies addressing different research questions. All participants had at least one fMRI (n=215, 60.9%) or EEG (n=260, 73.7%) assessment. Both fMRI and EEG were performed in 122 (34.6%) participants. The median [IQR] days between the CRS-R assessment and fMRI or EEG was 1 [0-2] and 0 [0-1] days, respectively. The CRS-R was performed within one day of fMRI or EEG in approximately 70% of participants, (Supplementary Appendix Figure S3). The demographic representativeness of our sample is addressed in Supplementary Appendix Table S5.

### Cognitive Motor Dissociation in Participants without Observable Response to Commands

Of 241 participants with a CRS-R diagnosis of coma/vegetative state (i.e., unconscious), or minimally conscious state minus, 60 (25%) responded to the command-following task on fMRI, EEG, or both (Figure 1). Supplementary Appendix Figures S4, S5 and Supplementary Appendix Table S6 provide the distribution of cognitive motor dissociation by CRS-R total score. Compared to participants without cognitive motor dissociation, participants with cognitive motor dissociation were younger (median [IQR] 30.5 [20.4] versus 45.3 [32.6] years), more likely to have a traumatic etiology (65% versus 38%), and more likely to have a CRS-R diagnosis of minimally conscious state minus (53% versus 38%). Participants with cognitive motor dissociation were also evaluated later post-injury or illness (10.7 [20.6] versus 4.3 [13.8] months, Table 2). Among participants with cognitive motor dissociation, 18% were assessed with fMRI only, 22% with EEG only, and 60% with both fMRI and EEG. The frequency of cognitive motor dissociation varied across sites (Supplementary Appendix Table S7). Supplementary Appendix Table S8 provides the proportion of participants with CRS-R diagnoses of coma/vegetative state and minimally conscious state minus who have positive and negative fMRI/EEG responses.

### Task-based fMRI and EEG responses in Participants with Observable Response to Commands

Of 112 participants with a CRS-R diagnosis of minimally conscious state plus or emerged from minimally conscious state, 43 (38%, Table 3) demonstrated command-following on task-based fMRI, task-based EEG, or both assessments. Among participants in this group, 23% were assessed with fMRI only, 19% with EEG only, and 58% with both fMRI and EEG. Responses to fMRI and EEG command-following tests were absent in more than 60% of participants who demonstrated evidence of command-following via behavioral responses on bedside assessment. Supplementary Appendix Table S9 provides the proportion of participants who demonstrated command-following on task-based fMRI or EEG stratified by CRS-R diagnosis, chronicity, and etiology.

Kappa coefficients indicating level of agreement between the behavioral diagnosis, fMRI, and EEG were low (0.09-0.15 for agreement between CRS-R and fMRI or EEG; 0.02-0.04 for agreement between fMRI and EEG; see Supplementary Appendix Tables S10 and S11).

## Discussion

In this multi-national investigation of a convenience sample of patients with disorders of consciousness, we detected cognitive motor dissociation on task-based fMRI or EEG in approximately 25%. This proportion is higher than previous estimates^3,6,9–11^. While standardized behavioral evaluation remains the reference standard for detecting command-following at the bedside, task-based fMRI and EEG can improve the detection rate, and performing both appears to be a more sensitive method.

The proportion of participants with cognitive motor dissociation in our study is 5-10 percent higher than previously reported.^3,6,9–11^ This finding may be due to our multi-modal approach, which classified participants based on responses to either fMRI or EEG in the 30% who had both assessments. The rate of cognitive motor dissociation may have been even higher if all participants were assessed with both modalities. Consistent with prior research, we found that cognitive motor dissociation is most common in patients with TBI,^3,6,9,11^ chronic disorders of consciousness,^9^ and a behavioral diagnosis of minimally conscious state minus.^11^ However, cognitive motor dissociation was also detected in participants with non-traumatic etiologies such as stroke and cardiac arrest, as well as in acute disorders of consciousness, and in patients who were behaviorally unconscious (coma/vegetative state).

The frequency of cognitive motor dissociation may be underestimated in prior studies and in ours for multiple reasons. First, the tasks used in active fMRI and EEG studies may require more cognitive resources (e.g., short term memory, selective attention, mental persistence) than typical command-following trials performed at the bedside. Although this hypothesis has not been proven (Supplementary Appendix Table S1)^33^, it is supported by our finding that fMRI and EEG responses were detected in only 38% of participants who demonstrated command-following behaviorally at the bedside. Second, the fMRI and EEG analytic techniques employed by the study sites are intentionally designed to minimize the potential for a false positive result, which may increase the likelihood of a false negative finding. Third, most studies assess participants with either fMRI or EEG. We found that participants assessed with both modalities were more likely to demonstrate cognitive motor dissociation. Finally, behavioral fluctuation is common in patients across all disorder of consciousness categories, which may contribute to negative fMRI or EEG findings or to disparate results between these two modalities.^34–36^

Several limitations should be considered when interpreting the results of this study. Participants were recruited using a variety of methods, including critically ill patients enrolled consecutively from the intensive care unit and those with chronic illness or injury enrolled by caregivers during the post-acute phase of recovery. All participants in the chronic group survived their initial illness or injury and had access to a research facility with advanced fMRI and EEG capabilities. This survival bias may reflect greater cognitive reserve and resilience over time. As such, our results may not be representative of global cognitive motor dissociation prevalence (see Supplementary Appendix Table S5). In the absence of standardized approaches to evaluate for cognitive motor dissociation, participating sites used heterogeneous strategies to acquire, analyze, and interpret data, leading to differences in the number, type, and ordering of the tasks. These differences, along with variations in recruitment strategies and participant characteristics, may contribute to the unequal proportion of cognitive motor dissociation observed at each site (ranging from 2% to 45%). Our findings may therefore not generalize across all centers. Large-scale validation studies are needed to optimize data acquisition and analysis for clinical translation. Statistical analyses conducted as part of this study were univariate and descriptive. Thus, we are unable to evaluate the independent contribution of any one variable in predicting cognitive motor dissociation. Agreement between cognitive motor dissociation detected by fMRI versus EEG was low which may result from fluctuations in awareness or differences in the underlying construct measured by each technique. Although participants were evaluated with CRS-R, fMRI, and EEG a variable number of times, for consistency, we analyzed the best performance from each modality and are unable to determine the number of assessments that were excluded due to poor performance. Serial CRS-R, fMRI, and EEG assessments may improve detection of cognitive motor dissociation but requires that these techniques be readily available. Finally, access to both the specially-trained personnel and technical assessments needed to assess for cognitive motor dissociation is presently available in only a few academic medical centers around the world, limiting the feasibility of performing these assessments in general practice.

Our results confirm, using neuroimaging and electrophysiologic methods that cognitive motor dissociation is more common than currently realized. Although task-based fMRI and EEG are not yet widely available for clinical assessment of disorders of consciousness, the knowledge that cognitive motor dissociation is not a rare occurrence should prompt further study to explore whether its detection can improve outcomes. Additionally, standardization, validation, and simplification of task-based fMRI and EEG methods used to detect cognitive motor dissociation is needed to prompt widespread clinical integration of these techniques and investigation of the bioethical implications of the findings.^37^

Disclosure forms provided by the authors are available with the full text of this article at NEJM.org.

## Supplementary Material

supplement

## Figures and Tables

**Table 1:** Participant Demographic and Injury Characteristics

Variable	Total Sample N=353^a^
Age at injury, median [IQR]	37.9 [23.8, 55.8]
Sex no. (%)	
Male	226 (64.4%)
Female	125 (35.6%)
Missing	2 (0.6%)
Months between injury and Coma Recovery Scale-Revised assessment, median [IQR]	7.9 [1.0, 22.1]
< 28 days post injury no. (%)	90 (25.5%)
Etiology no. (%)	
Traumatic brain injury	176 (49.9%)
Cardiac arrest/anoxia	57 (16.1%)
SAH, IVH, ICH, stroke, aneurysm	65 (18.4%)
Other	55 (15.6%)
Diagnosis (based on the Coma Recovery Scale-Revised), no. (%)	
Unconscious (coma/vegetative state)	140 (39.7%)
Minimally conscious state minus	101 (28.6%)
Minimally conscious state plus	77 (21.8%)
Emerged from minimally conscious state	35 (9.9%)

a all proportions are calculated from the number of participants indicated in the column heading (n=353); Minimally conscious state minus = minimally conscious state without command-following, Minimally conscious state plus = minimally conscious with command-following

**Abbreviations**: ICH *intracerebral hemorrhage*; IVH *intraventricular hemorrhage*; SAH *subarachnoid hemorrhage*

**Table 2:** Demographics, Clinical Characteristics, and fMRI/EEG Results in Participants Without Observable Command-following

Variable	All Participants Without Observable Command-following N=241^a^	+fMRI or +EEG (i.e., cognitive motor dissociation) N=60	-fMRI and -EEG N=181
Diagnosis (based on the Coma Recovery Scale-Revised), no. (%)			
	140 (58.1%)	28 (46.7%)	112 (61.9%)
Unconscious (coma/vegetative state)	101 (41.9%)	32 (53.3%)	69 (38.1%)
Minimally conscious state minus			
Assessed with fMRI only no. (%)	61 (25.3%)	11 (18.3%)	50 (27.6%)
Assessed with EEG only no. (%)	101 (41.9%)	13 (21.7%)	88 (48.6%)
Assessed with fMRI and EEG no. (%)	79 (32.8%)	36 (60.0%)	43 (23.8%)
Age at injury, median [IQR]	40.2 [15.0]	30.5 [20.4]	45.3 [32.6]
Sex no. (%)			
Male	146 (60.6%)	39 (65.0%)	107 (59.1%)
Female	93 (38.6%)	21 (35.0%)	72 (39.8%)
Missing	2 (0.8%)	0 (0%)	2 (1.1%)
Months between injury and Coma Recovery Scale-Revised assessment, median [IQR]	6.3 [16.3]	10.7 [20.6]	4.3 [13.8]
< 28 days post injury/illness no. (%)	72 (29.9%)	12 (20.0%)	60 (33.1%)
≥ 28 days post injury/illness no. (%)	169 (70.1)	48 (80.0%)	121 (66.9%)
Etiology no. (%)			
Traumatic brain injury	108 (44.8%)	39 (65.0%)	69 (38.1%)
Cardiac arrest/anoxia	45 (18.6%)	4 (6.7%)	41 (22.7%)
SAH, IVH, ICH, stroke, aneurysm	48 (19.9%)	9 (15.0%)	39 (21.6%)
Other	40 (16.6%)	8 (13.3%)	32 (17.7%)

a all proportions are calculated from the number of participants indicated in the column heading; for example, of 241 participants with a Coma Recovery Scale-Revised (CRS-R) behavioral diagnosis of coma or vegetative state (unconscious) or minimally conscious state minus (minimally conscious state without command-following), 140 (58.1%) were unconscious. “+fMRI or +EEG” indicates that at least one assessment (either fMRI or EEG regardless of whether participants had one or both of these assessments) was positive. “-fMRI and -EEG” indicates that for participants with fMRI only, the fMRI assessment was negative; for participants with EEG only, the EEG assessment was negative; for participants with both fMRI and EEG, both assessments were negative.

**Abbreviations**: ICH *intracerebral hemorrhage*; IVH *intraventricular hemorrhage*; EEG *electroencephalography*; fMRI *functional magnetic resonance imaging*; SAH *subarachnoid hemorrhage*

**Table 3:** Demographics, Clinical Characteristics, and fMRI/EEG Results in Participants With Observable Command-following

Variable	All Participants With Observable Command-following N=112^a^	+fMRI or +EEG N=43	-fMRI and -EEG N=69
Diagnosis (based on the Coma Recovery Scale-Revised) no. (%)	77 (68.8%) 35 (31.3%)	26 (60.5%) 17 (39.5%)	51 (73.9%) 18 (26.1%)
Minimally conscious state plus			
Emerged from the minimally conscious state			
Assessed with fMRI only no. (%)	32 (28.6%)	10 (23.3%)	22 (31.9%)
Assessed with EEG only no. (%)	37 (33.0%)	8 (18.6%)	29 (42.0%)
Assessed with fMRI and EEG no. (%)	43 (38.4%)	25 (58.1%)	18 (26.1%)
Age at injury, median [IQR]	33.8 [32.4]	29.4 [24.7]	38.6 [33.0]
Sex no. (%)			
Male	80 (71.4%)	30 (69.8%)	50 (72.5%)
Female	32 (28.6%)	13 (30.2%)	19 (27.5%)
Months between injury and Coma Recovery Scale-Revised assessment, median [IQR]	12.9 [45.3]	12.6 [51.9]	12.9 [40.7]
< 28 days post injury/illness no. (%)	18 (16.1%)	10 (23.3%)	8 (11.6%)
≥ 28 days post injury/illness no. (%)	94 (83.9%)	33 (76.7%)	61 (88.4%)
Etiology no. (%)			
Traumatic brain injury	68 (60.7%)	30 (69.8%)	38 (55.1%)
Cardiac arrest/anoxia	12 (10.7%)	1 (2.3%)	11 (15.9%)
SAH, IVH, ICH, stroke, aneurysm	17 (15.2%)	9 (20.9%)	8 (11.6%)
Other	15 (13.4%)	3 (7.0%)	12 (17.4%)

a all proportions are calculated from the number of participants indicated in the column heading; for example, of 112 patients with a Coma Recovery Scale-Revised (CRS-R) behavioral diagnosis of minimally conscious state plus (minimally conscious state with command-following) or emerged from the minimally conscious state, 77 (68.8%) had a CRS-R diagnosis of minimally conscious state plus. “+fMRI or +EEG” indicates that at least one assessment (either fMRI or EEG regardless of whether participants had one or both of these assessments) was positive. “-fMRI and -EEG” indicates that for participants with fMRI only, the fMRI assessment was negative; for participants with EEG only, the EEG assessment was negative; for participants with both fMRI and EEG, both assessments were negative.

**Abbreviations**: ICH *intracerebral hemorrhage*; IVH *intraventricular hemorrhage*; EEG *electroencephalography*; fMRI *functional magnetic resonance imaging*; SAH *subarachnoid hemorrhage*; TBI *traumatic brain injury*
