# Supplementary material for: Cognitive Motor Dissociation in Disorders of Consciousness
Source: N Engl J Med. Author manuscript; Available in PMC 2024 Dec 10. (PMC7617195; doi:10.1056/NEJMoa2400645)
Supplement: supplement [file EMS197475-supplement-supplement.pdf]

Supplementary Appendix to:

Cognitive Motor Dissociation in Disorders of Consciousness

Yelena G Bodien PhD[1,2,3,4], Judith Allanson, FRCP PhD[5,6], Paolo Cardone MS[7,8], Arthur Bonhomme BS[7,8], Jerina Carmona MPH[9], Camille Chatelle PhD [2,4,7,8,10], Srivas Chennu PhD[6,11,12], Mary Conte PhD[13], Stanislas Dehaene PhD[14], Paola Finoia PhD[11], Gregory Heinonen BS[9], Jennifer Hersh[13,15], Evelyn Kamau[6,11], Phoebe Lawrence BS[2], Victoria C. Lupson[6,16], Anogue Meydan BS[2], Benjamin Rohaut MD PhD[17,18], William R Sanders BS[2], Jacobo D Sitt MD PhD[17], Andrea Soddu PhD[19,20], Mélanie Valente[17,18], Angela Velazquez[9], Henning U Voss PhD[21], Athina Vrosgou MSc[9], Jan Claassen MD[9], Brian L Edlow MD[2,4,22], Joseph J Fins MD[15,23,24], Olivia Gosseries PhD[7,8], Steven Laureys MD PhD[7,25,26], David Menon MD[16,27], Lionel Naccache MD PhD[17,18], Adrian M Owen PhD[20], John Pickard MChir[6,11,16], Emmanuel A Stamatakis PhD[6,27], Aurore Thibaut PhD[7,8], Jonathan D Victor MD PhD[13,23,28], Joseph T Giacino PhD[1,3], Emilia Bagiella PhD[29], Nicholas D Schiff MD[13,23,28]

Affiliations:
[1]Department of Physical Medicine and Rehabilitation, Spaulding Rehabilitation Hospital, Charlestown MA, USA, [2]Center for Neurotechnology and Neurorecovery, Department of Neurology, Massachusetts General Hospital, Boston MA, USA, [3]Department of Physical Medicine and Rehabilitation, Harvard Medical School, Cambridge MA, USA, [4]Department of Neurology, Harvard Medical School, Cambridge MA, USA, [5]Department of Neurosciences, Addenbrookes Hospital, Cambridge UK, [6]Department of Clinical Neurosciences, University of Cambridge, UK, [7]Coma Science Group, GIGA Consciousness Research Unit, University and University Hospital of Liège, Liège, Belgium, [8]Centre du Cerveau, University Hospital of Liège, Liège, Belgium, [9]Department of Neurology, Columbia University Irving Medical Center, Presbyterian Hospital, New York, NY, USA, [10]European Research Council Executive Agency, Brussels, Belgium*, [11]Division of Neurosurgery, School of Clinical Medicine, University of Cambridge, Cambridge, UK, [12]School of Computing, University of Kent, Kent, UK, [13]Feil Family Brain and Mind Research Institute, Weill Cornell Medicine, New York, NY, USA, [14]Collège de France, Université Paris-Sciences-Lettres, Paris, France, [15]Division of Medical Ethics, Weill Cornell Medicine, New York, NY, USA, [16]Wolfson Brain Imaging Centre, University of Cambridge, Cambridge, UK, [17]Sorbonne Université, Institut du Cerveau—Paris Brain Institute—ICM, INSERM, CNRS, Paris, France, [18]AP-HP, Hôpital Pitié-Salpêtrière, DMU Neurosciences, Paris, France, [19]Physics & Astronomy Department, University of Western Ontario, London ON, Canada, [20]Western Institute for Neuroscience, University of Western Ontario, London ON, Canada, [21]Department of Radiology, Weill Cornell Medicine, New York, NY, USA, [22]Athinoula A. Martinos Center for Biomedical Imaging, Massachusetts General Hospital, Charlestown, MA, USA, [23]The Rockefeller University Hospital, New York, NY, USA, [24]Yale Law School, New Haven, CT, [25]CERVO Brain Research Centre 2601, de la Canardière, Québec Canada G1J 2G3, [26]Consciousness Science Institute, Hangzhou Normal University, Hangzhou, Zhejiang, China. [27]Division of Anaesthesia, Department of Medicine, University Cambridge, Cambridge, UK,[28]Department of Neurology, Weill Cornell Medicine, New York, NY, USA, [29]Department of Population Health Science & Policy, Center for Biostatistics, Icahn School of Medicine at Mount Sinai, New York, NY, USA

*The views expressed are purely those of the writer and may not in any circumstances be regarded as stating an official position of the European Research Council Executive Agency (ERCEA) and the European Commission.

Table of Contents	Page
Figure S1: Description of the Sample and Inclusion of Participants in Prior Publications by Site	3
Figure S2: Months between Injury/Illness and CRS-R Assessment	4
Figure S3: Days Between CRS-R Assessment, fMRI, and EEG	5
Figure S4: CRS-R Total Scores in Participants Stratified by fMRI and EEG Responses	6
Figure S5: CRS-R Total Scores in Participants Stratified by CRS-R Diagnosis	7
Table S1: Comparing Behavioral and fMRI/EEG Approaches to Assessment of Command-Following	8
Table S2: Relevant References that Contain Detailed Methodology Adopted by Each Site	9
Table S3: Summary of fMRI and EEG Key Design Elements for Each Site	11
Table S4: CRS-R Behaviors and Diagnostic Categories	12
Table S5: Representativeness of the Sample	13
Table S6: CRS-R Total Scores in Participants Stratified by fMRI and EEG Responses	14
Table S7: Cognitive Motor Dissociation Proportions by Site	15
Table S8: CRS-R Diagnosis, Assessment Method, Chronicity, and Etiology Stratified by fMRI and EEG Responses in Participants Without Observable Command-Following	16
Table S9: CRS-R Diagnosis, Assessment Method, Chronicity, and Etiology Stratified by fMRI and EEG Responses in Participants Without Observable Command-Following	17
Table S10: Concordance Between Command-Following on the CRS-R, fMRI, and EEG	18
Table S11: Concordance Between Covert Command-Following on fMRI and EEG	19
Supplementary References	20

Figure S1: Description of the Sample and Inclusion of Participants in Prior Publications by Site

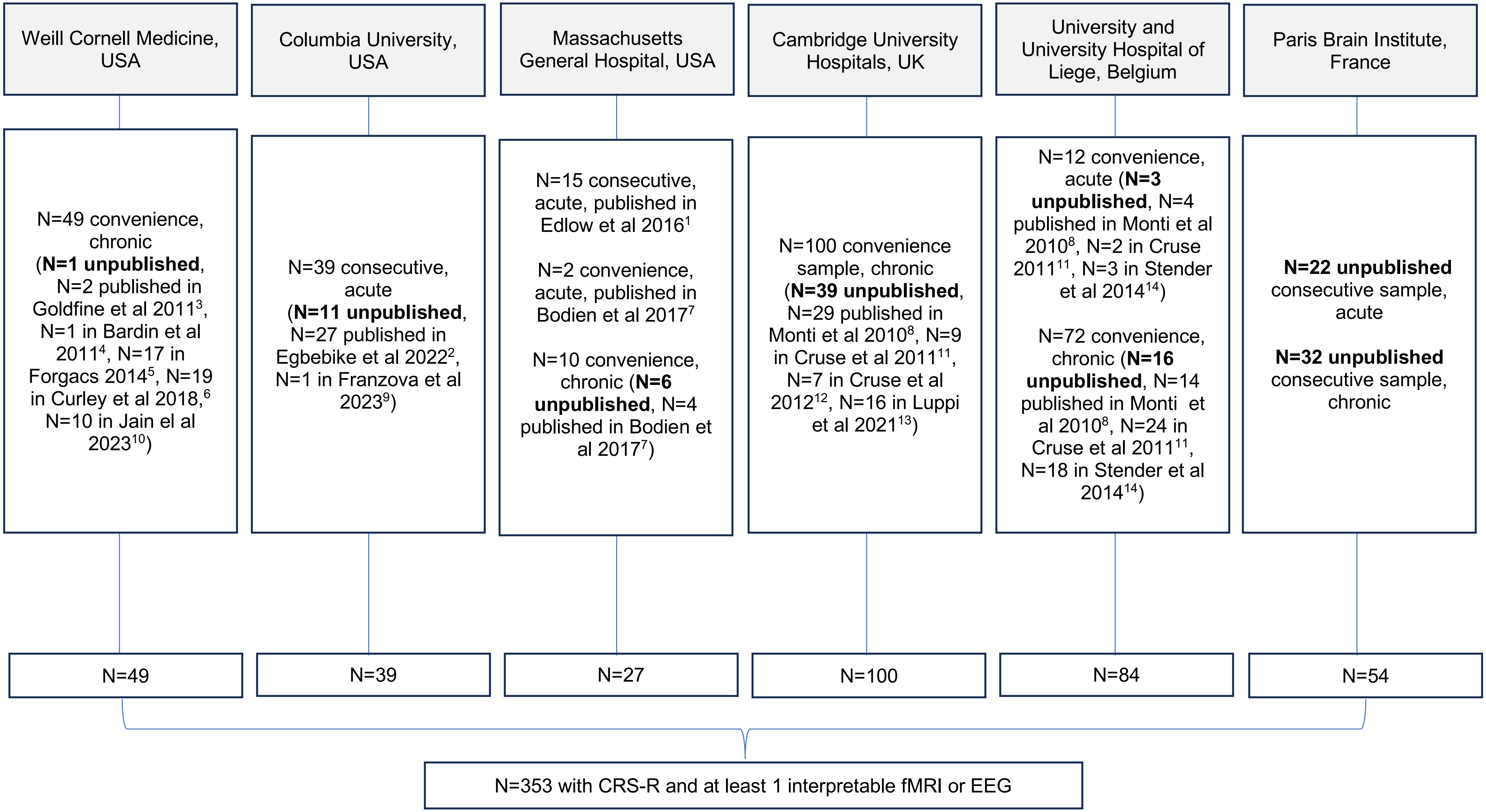

Description of participants from each site contributing to the sample of 353 with one CRS-R standardized behavioral assessment and one task-based fMRI and/or EEG assessment. Each site enrolled all participants prospectively and adhered to local practices for maintaining screening logs. Participants included in prior publications were typically part of a larger sample aimed at answering a variety of questions related to disorders of consciousness.
Abbreviations: CRS-R *Coma Recovery Scale-Revised;* EEG *electroencephalography;* fMRI *functional magnetic resonance imaging*

Figure S2: Months between Injury/Illness and CRS-R Assessment

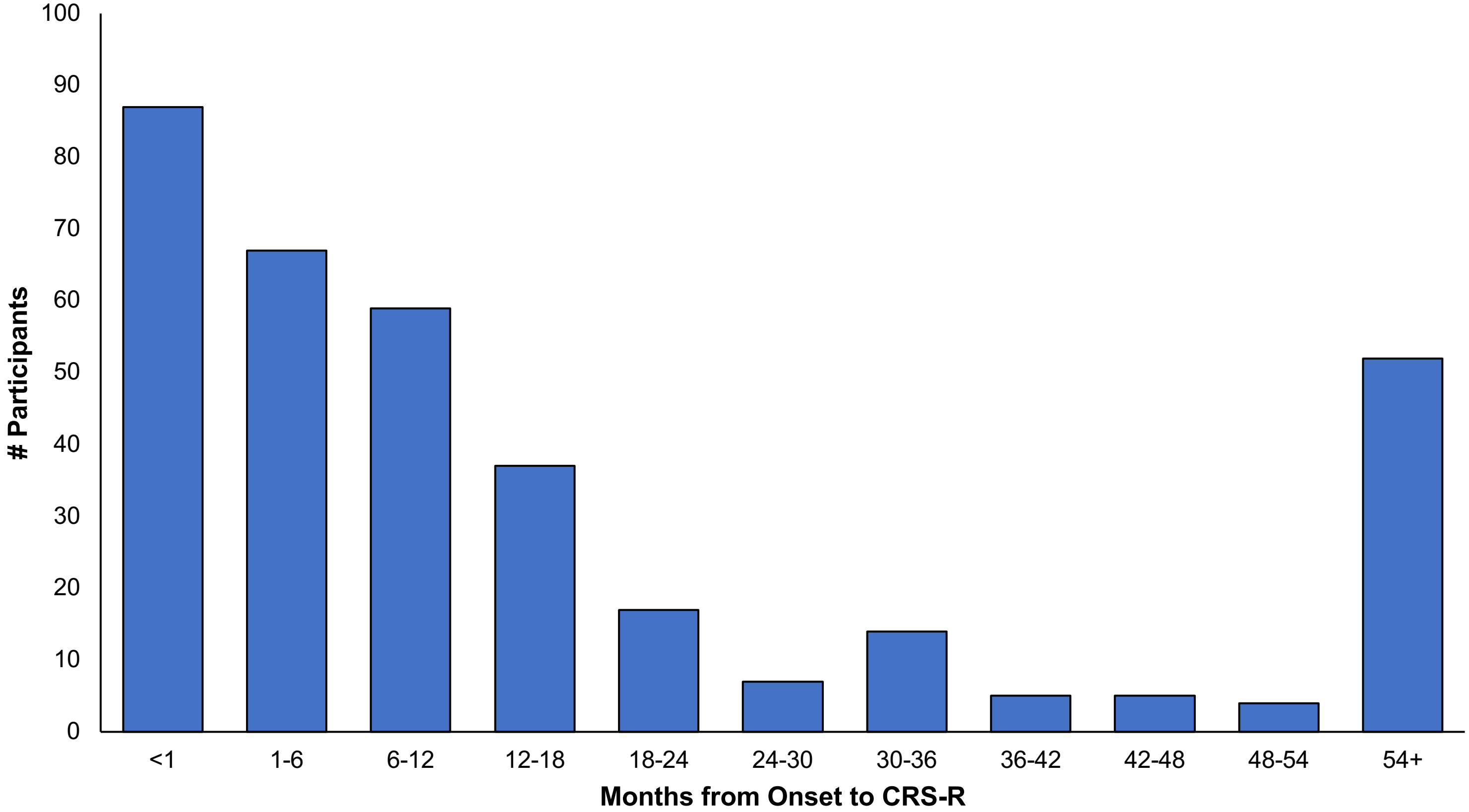

Approximately 25% of participants were in the acute stage of recovery at time of evaluation (<1 month post-injury/illness onset). Participants in the chronic stage of recovery were typically within two years or more than 5 years post injury/illness onset.

Abbreviations: CRS-R *Coma Recovery Scale-Revised*

Figure S3: Days Between CRS-R Assessment, fMRI, and EEG

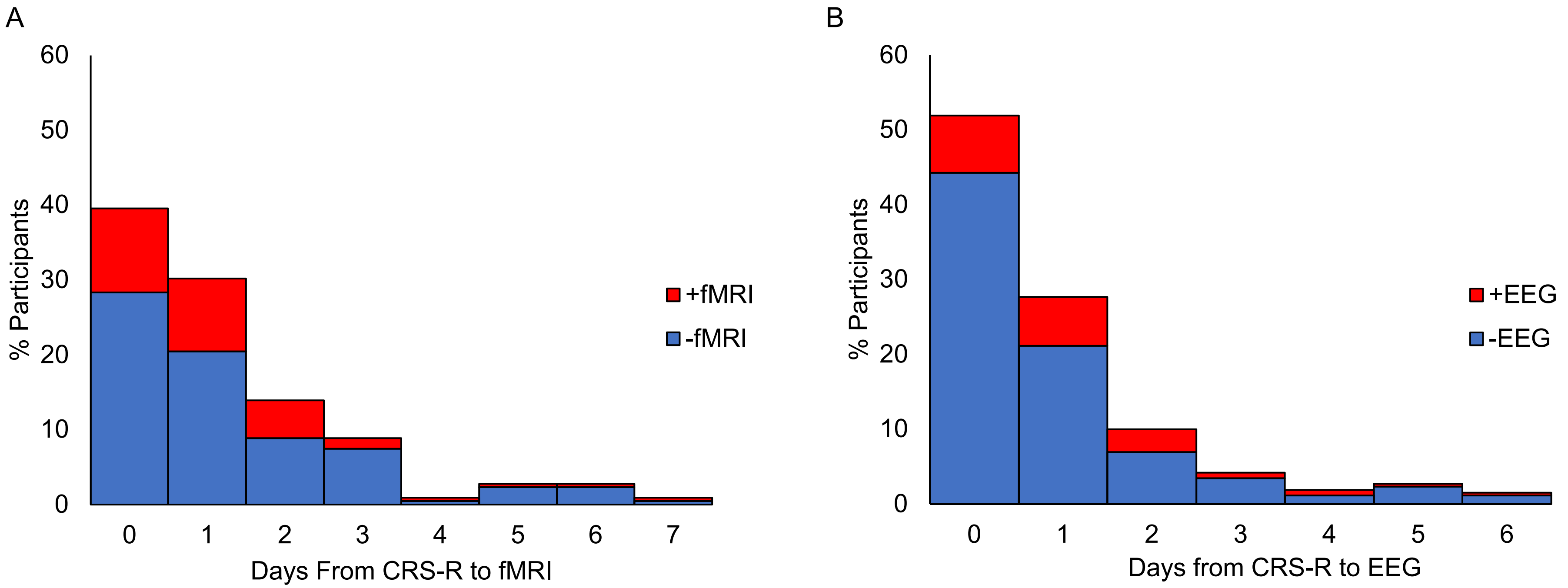

fMRI (A, 215 MRI scans) and EEG (B, 260 EEG acquisitions) data were typically acquired within 0-1 days of the CRS-R to minimize the effect of fluctuations on detection on CMD. +fMRI or +EEG indicates a response to task-based fMRI or EEG was detected, and -fMRI or -EEG indicates a response to task-based fMRI or EEG was not detected

Abbreviations: CRS-R *Coma Recovery Scale-Revised*; EEG *electroencephalography*; fMRI *functional magnetic resonance imaging*

Figure S4: CRS-R Total Scores in Participants Stratified by fMRI and EEG Responses

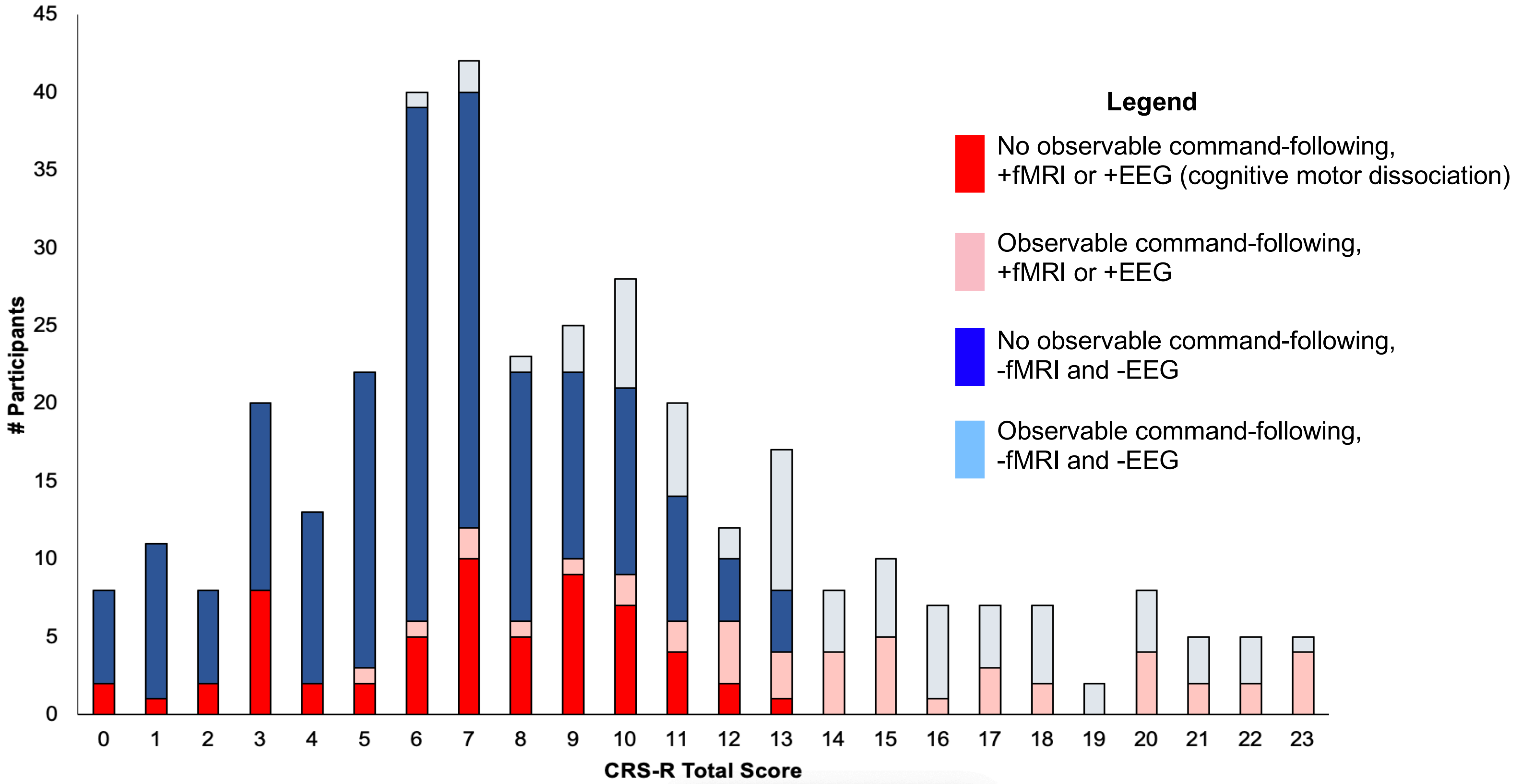

The number of participants with each CRS-R total score is stratified by positive responses (red) and negative responses (blue) to task-based fMRI and/or EEG. Saturated red and blue bars represent participants with no observable command-following on the CRS-R (i.e., diagnosis of coma/VS [unconscious] or minimally conscious state minus [minimally conscious state without command-following) while opaque red and blue bars represent participants with observable command-following on the CRS-R (i.e., diagnosis of minimally conscious state plus [minimally conscious state with command-following] or emerged from minimally conscious state). "+fMRI or +EEG" indicates that at least one assessment (either fMRI or EEG regardless of whether participants had one or both of these assessments) was positive. "-fMRI and -EEG" indicates that all assessments (fMRI, EEG, or both fMRI and EEG for participants who had both assessments) were negative. Cognitive motor dissociation (red saturated bar) is most common in participants with CRS-R total score in the 7-10 range but is also present across the full range of CRS-R scores that are associated with a diagnosis of coma, vegetative state, or minimally conscious state minus (i.e., total scores 0-13).

Abbreviations: CRS-R *Coma Recovery Scale*-Revised; EEG *electroencephalography;* fMRI *functional magnetic resonance imaging*

Figure S5: CRS-R Total Scores in Participants Stratified by CRS-R Diagnosis

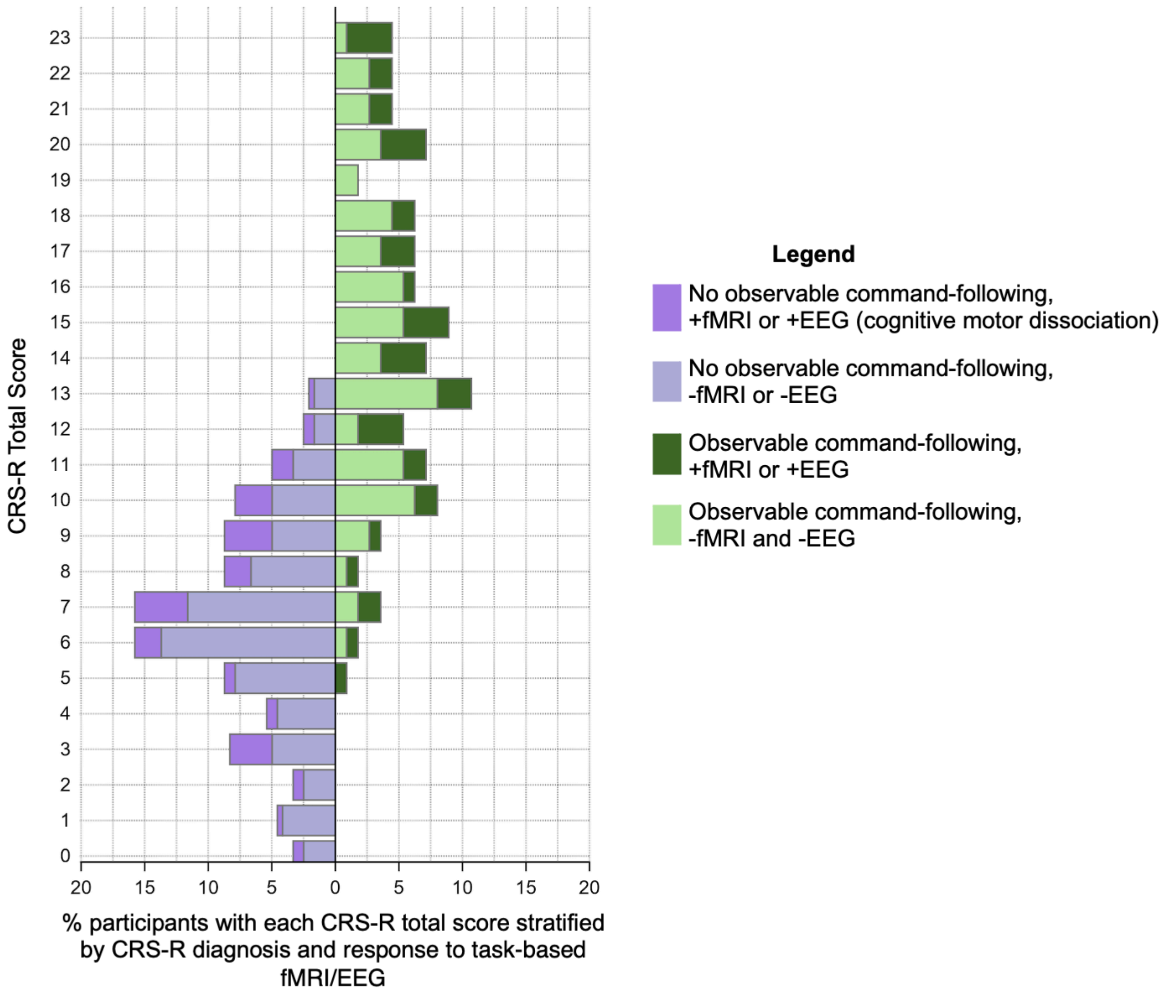

The proportion of participants with each CRS-R total score is stratified by participants with no observable command-following on the CRS-R (purple, diagnosis of coma/vegetative state (unconscious) or minimally conscious state minus (minimally conscious state without command-following) and participants with observable command-following on the CRS-R (green, diagnosis of minimally conscious state plus [minimally conscious state with command-following] or emerged from minimally conscious state). Saturated purple and green bars represent participants with +fMRI or +EEG while opaque purple and green bars represent participants with -fMRI and -EEG. "+fMRI or +EEG" indicates that at least one assessment (either fMRI or EEG regardless of whether participants had one or both of these assessments) was positive. "-fMRI and -EEG" indicates that all assessments (fMRI, EEG, or both fMRI and EEG for participants who had both assessments) were negative.

Abbreviations: CRS-R *Coma Recovery Scale*-Revised; EEG *electroencephalography;* fMRI *functional magnetic resonance imaging*

Table S1: Comparing Behavioral and fMRI/EEG Approaches to Assessment of Command-Following

Approach to Assessment of Command-Following	Example Command	Cognitive Functions Required	Minimum Number of Correct Responses	Response Window	Duration of Assessment
Clinical-bedside	Wiggle your toes	• primary auditory/visual processing<br>• language comprehension<br>• working memory<br>• motor planning<br>• initiation<br>• motor execution	1	Not specified	seconds
Standardized-bedside<br>e.g., Coma Recovery Scale-Revised	Wiggle your toes	• primary auditory/visual processing<br>• language comprehension<br>• working memory<br>• motor planning<br>• initiation<br>• motor execution<br>• cognitive/motor persistence<br>• sustained attention	3 out of 4 trials	Within 10 seconds of command	~2 minutes
Task-based fMRI/EEG	Imagine wiggling your toes	• primary auditory/visual processing<br>• language comprehension<br>• working memory<br>• short term memory<br>• capacity to recruit schematic representation<br>• kinesthetic proprioceptive awareness<br>• motor planning+<br>• initiation+<br>• cognitive/motor persistence+<br>• sustained attention+	Continuous responses for multiple 15-30 second periods	Immediately following command until command to "stop"	≥ 5 minutes

Command-following detected by task-based functional magnetic resonance imaging (fMRI) and electroencephalography (EEG) requires more cognitive functions and has an increased cognitive load compared to standardized bedside assessment, which in turn requires more cognitive functions and has an increased cognitive load compared to a clinical bedside assessment. A "+" identifies cognitive functions that are required across multiple approaches to assessing command-following but are required to a greater extent for command-following on task-based fMRI/EEG.

Table S2: Relevant References that Contain Detailed Methodology Adopted by Each Site

Site	Author	Year	Journal	Title	DOI	fMRI or EEG methods
Weill Cornell Medicine, New York USA	Bardin et al.[4]	2011	Brain	Dissociations between behavioural and functional magnetic resonance imaging-based evaluations of cognitive function after brain injury	10.1093/brain/awr005	fMRI
	Goldfine et al.[3]	2011	Clinical Neurophysiology	Determination of awareness in patients with severe brain injury using EEG power spectral analysis	10.1016/j.clinph.2011.03.022	EEG
	Goldfine et al.[15]	2013	Lancet	Reanalysis of "Bedside detection of awareness in the vegetative state: a cohort study"	10.1016/S0140-6736(13)60125-7	
	Forgacs et al.[5]	2014	Annals of Neurology	Preservation of electroencephalographic organization in patients with impaired consciousness and imaging-based evidence of command-following	10.1002/ana.24283	
	Curley et al.[16]	2018	Brain	Characterization of EEG signals revealing covert cognition in the injured brain	10.1093/brain/awy070	
Columbia University Irving Medical Center, New York USA	Claassen, et al[17]	2019	NEJM	Detection of brain activation in unresponsive patients with acute brain injury	10.1056/NEJMoa1812757	EEG
Massachusetts General Hospital, Boston USA	Edlow, et al[1]	2017	Brain	Early detection of consciousness in patients with acute severe traumatic brain injury	10.1093/brain/awx176	fMRI/EEG

Cambridge University Hospitals NHS Trust, UK	Luppi, et al[13]	2021	NeuroImage: Clinical	Preserved fractal character of structural brain networks is associated with covert consciousness after severe brain injury	10.1016/j.nicl.2021.102682	fMRI
	Monti, et al[8]	2010	NEJM	Willful modulation of brain activity in disorders of consciousness	10.1056/NEJMoa0905370	
	Cruse, et al[11]	2011	Lancet	Bedside detection of awareness in the vegetative state: a cohort study	10.1016/S0140-6736(11)61224-5.	EEG
University and University Hospital of Liege, Belgium	Monti, et al[8]	2010	NEJM	Willful modulation of brain activity in disorders of consciousness	10.1056/NEJMoa0905370	fMRI
	Cruse, et al[11]	2011	Lancet	Bedside detection of awareness in the vegetative state: a cohort study	10.1016/S0140-6736(11)61224-5.	EEG
	Lule et al[18]	2013	Clin Neurophysiol.	Probing command following in patients with disorders of consciousness using a brain-computer interface	10.1016/j.clinph.2012.04.030	
	Lesenfants, et al[19]	2018	Clin EEG Neurosci	Toward an attention-based diagnostic tool for patients with locked-in syndrome	10.1177/1550059416674842	
Paris Brain Institute, France	Claassen, et al[17]	2019	NEJM	Detection of brain activation in unresponsive patients with acute brain injury	10.1056/NEJMoa1812757	EEG

The references in this table include the methodologic details for functional magnetic resonance imaging (fMRI) and electroencephalography (EEG) acquisitions and interpretations used to determine our findings. In some cases, participants in our study were co-enrolled in the studies listed in this table.

Table S3: Summary of fMRI and EEG Key Design Elements for Each Site

Site	Patients (N)	Inclusion[a]	fMRI[b]		EEG[c]			
			Head Coil	Paradigm	Electrode Type	Electrode Count	Filter Setting	Paradigm
Weill Cornell Medicine, USA	49	Chronic	32 ch	• Hand (act,img)<br>• Tennis (img)<br>• Swim (img)<br>• Spatial navigation (img)	Collodian Pasted	37	.1/.3-100 Hz Bandpass	• Tennis (img)<br>• Swim (img)
Columbia University, USA	39	Acute			Collodian Pasted	21	1-70 Hz Bandpass	Hand (act)
Massachusetts General Hospital, USA	27	Acute, chronic, TBI only	32 ch	Hand (img)	Collodian Pasted	19	1-30 Hz Butterworth	Hand (img)
Cambridge University Hospitals, UK	100	Chronic	12 ch	• Tennis (img)<br>• Spatial navigation (img)<br>• Communication	Geodesic Net	25 (C3-C4) of 129	1-40 Hz Notch	• Hand (act)<br>• Toes (act)
University and University Hospital of Liege, Belgium	84	Acute, chronic	32 ch	• Tennis (img)<br>• Spatial navigation (img)	EGI Water-based	25 (C3-C4) of 129	1-40 Hz Bandpass	• Hand (act)<br>• Toes (act)<br>• Counting targets<br>• Attending to color
Paris Brain Institute, France	54	Acute, chronic			High-density Cap	250	0.5 and 20 Hz Bandpass	Hand (act)

[a] Across all sites, primary inclusion criteria were: adults ≥18 years of age (some sites excluded participants ≥75, 76 or 80 years of age), behavioral diagnosis of vegetative state or minimally conscious state, family/surrogate consent for enrollment. Some sites also included patients emerged from the minimally conscious state. For sites enrolling acutely (i.e., <28 days post injury/illness,) patients in coma were also included and patients with deep sedation were excluded. All sites excluded patients with prior neurological or psychiatric disease and contraindication for MRI/EEG (as appropriate based on modalities used at each site, e.g. for fMRI, ability to lay flat for at least an hour and no ferrous metal implants). Sites that required patients to travel to the clinical/research center excluded patients who could not tolerate travel. Additional exclusion criteria present at a single site were: ventilator dependence, dialysis dependence, significant acute or chronic medical illness, participation in any investigational trial within 30 days.
[b] When multiple fMRI assessments were available, we analyzed the first assessment. All sites used a 3T MRI scanner and a region of interest (ROI) approach for data analysis. All paradigms involved an active (act, e.g., open and close your hand) or imagined (img, imagine opening and closing your hand) command. For one site, Cambridge University Hospitals, the paradigm was based on communicating yes/no responses to questions by attending to the spoken words "yes" and "no".
[c] When multiple EEG assessments were available, we analyzed the first assessment. EEG analytic approaches varied (see Table S2)

Abbreviations*: act* motor action paradigm, ch channels*, EEG* electroencephalography, *fMRI* functional magnetic resonance imaging, *img* imagery paradigm, NY *New York, USA*, TBI *traumatic brain injury*, UK *United Kingdom.* Study design elements are mapped to Disorders of Consciousness Common Data Elements when possible. (see Carroll et al. *Neurocritical Care* 2023 for EEG and Edlow et al. *Neurocritical Care* 2023 for fMRI).

Table S4: CRS-R Behaviors and Diagnostic Categories

AUDITORY FUNCTION SCALE	OROMOTOR/VERBAL FUNCTION SCALE
4 – Consistent Movement to Command§	3 – Intelligible Verbalization§
3 – Reproducible Movement to Command§	2 – Vocalization/Oral Movement
2 – Localization to Sound	1 – Oral Reflexive Movement
1 – Auditory Startle	0 – None
0 – None	**COMMUNICATION SCALE**
**VISUAL FUNCTION SCALE**	2 – Functional: Accurate†
5 – Object Recognition§	1 – Non-functional: Intentional§
4 – Object Localization: Reaching*	0 – None
3 – Visual Pursuit*	**AROUSAL SCALE**
2 – Fixation*	3 – Attention
1 – Visual Startle	2 – Eye Opening w/o Stimulation
0 – None	1 – Eye Opening with Stimulation
**MOTOR FUNCTION SCALE**	0 – Unarousable
6 – Functional Object Use†	* Denotes Minimally Conscious State minus (MCS-)<br>§ Denotes Minimally Conscious State plus (MCS+)<br>† Denotes Emergence from Minimally Conscious State (eMCS)
5 – Automatic Motor Response*	
4 – Object Manipulation*	
3 – Localization to Noxious Stimulation*	
2 – Flexion Withdrawal	
1 – Abnormal Posturing	
0 – None	

In the vegetative state (also known as unresponsive wakefulness syndrome), there is no behavioral evidence of consciousness. The minimally conscious state can be subdivided into minimally conscious state without command-following and intelligible speech (MCS-; e.g., visual pursuit) and with command-following or intelligible speech (MCS+). These behaviors often fluctuate over minutes to hours. Finally, emergence from minimally conscious state indicates recovered consciousness although disorientation, lack of awareness for current circumstances, cognitive impairments, and other clinical symptoms are often present.

When multiple Coma Recovery Scale-Revised (CRS-R) assessments were available, we used the CRS-R corresponding to the highest level of consciousness documented within 7 days of fMRI and EEG.

Table S5: Representativeness of the Sample

**Category**	**Description**
Condition under investigation	Disorders of consciousness
**Considerations related to**	
Sex	In prior published studies, disorders of consciousness were observed in males more than females (1.3:1 - 2:1).[20-25]
Age	Disorders of consciousness can affect individuals across the age spectrum, from pediatric to geriatric.[20,21,23-27]
Race or ethnic group	More than 50% of participants in this study were enrolled in the UK and European countries, where information on race and ethnicity are not routinely acquired. Therefore, these characteristics were not part of the minimum dataset included in our centralized REDCap repository.
Geography	Published data on disorders of consciousness are predominantly available from the US, UK, and Europe, which is consistent with the geographic distribution of the cohort in this study. Disorders of consciousness have also been studied in South America[28,29] and Asia,[30,31] where reported age and sex characteristics are similar to our cohort.
Other considerations	There are no large-scale, systematic epidemiological studies of disorders of consciousness that can inform the demographic characteristics of this condition. The lack of this information is due, in part, to the absence of medical codes that precisely differentiate between states of consciousness during the acute stage of recovery and systematic long-term assessment in the chronic stage of recovery. Figures in this table are based on demographic information published in individual studies and meta-analyses.
Overall representativeness of this trial	Systematic, global studies on the prevalence and characteristics of persons with disorders of consciousness are not available.[32,33] Prior publications are biased due to approach to sampling (e.g., convenience sample requiring consent), limiting enrollment to a single site, or evaluating a single etiology. The participants in this study demonstrate the expected distribution of sex given that published studies consistently report more males than females in studies of disorders of consciousness.[20-24] We report data on sex and not on gender identity because questions regarding gender identity were not commonly asked for the majority of the past 15-year period during which data for this study were collected. Moreover, participants in this study cannot communicate and therefore cannot provide self-report information regarding gender identity. The age of our sample is consistent with prior studies of adults.[20,21,23-26] Disorders of consciousness are also present in pediatric patients;[27] however, our cohort only includes adults, and our findings may not generalize to children. Consistent with prior studies of disorders of consciousness, and many studies conducted in Europe and the UK, we do not report the race and ethnicity of our sample. Given the lack of data on disorders of consciousness in racial and ethnic minorities, low- and middle-income countries, or even in regions outside of select academic centers, it is unknown whether our study is representative beyond the regions and demographics in which the data were acquired.

Table S6: CRS-R Total Scores in Participants Stratified by fMRI and EEG Responses

CRS-R Total	+fMRI or +EEG		-fMRI and -EEG	
	No observable command-following on CRS-R [coma/vegetative state, minimally conscious state minus] (cognitive motor dissociation) %, out of 103 [95% CI]	Observable command-following on CRS-R [minimally conscious state plus, emerged from minimally conscious state] %, out of 103 [95% CI]	No observable command-following on CRS-R [coma/vegetative state, minimally conscious state minus] %, out of 250 [95% CI]	Observable command-following on CRS-R [minimally conscious state plus, emerged from minimally conscious state] %, out of 250 [95% CI]
0	1.9 [0.34, 7.52]		2.4 [0.98, 5.40]	
1	1.0 [0.05, 6.07]		4.0 [2.05, 7.46]	
2	1.9 [0.34, 7.52]		2.4 [0.98, 5.40]	
3	7.8 [3.66, 15.2]		4.8 [2.62,8.45]	
4	1.9 [0.34, 7.52]		4.4 [2.33, 7.95]	
5	1.9 [0.34, 7.52]	1.0 [0.05, 6.07]	7.6 [4.76, 11.80]	
6	4.9 [1.80, 11.50]	1.0 [0.05, 6.07]	13.2 [9.39, 18.20]	0.4 [0.02, 2.56]
7	9.7 [5.01, 17.50]	1.9 [0.34, 7.52]	11.2 [11.0, 11.70]	0.8 [0.14, 3.17]
8	4.9 [1.80, 11.50]	1.0 [0.05, 6.07]	6.4 [3.82, 10.40]	0.4 [0.02, 2.56]
9	8.7 [4.32, 16.40]	1.0 [0.05, 6.07]	4.8 [2.62, 8.45]	1.2 [0.31, 3.76]
10	6.8 [3.01, 14]	1.9 [0.34, 7.52]	4.8 [2.62, 8.45]	2.8 [1.23, 5.93]
11	3.9 [1.25, 10.20]	1.9 [0.34, 7.52]	3.2 [1.50, 6.44]	2.4 [0.98, 5.40]
12	1.9 [0.34, 7.52]	3.9 [1.25, 10.20]	1.6 [0.51, 4.32]	0.8 [0.14, 3.17]
13	1.0 [0.05, 6.07]	2.9 [0.76, 8.90]	1.6 [0.51, 4.32]	3.6 [1.77, 6.95]
14		3.9 [1.25, 10.20]		1.6 [0.51, 4.32]
15		3.9 [1.25, 10.20]		2.4 [0.98, 5.40]
16		1.0 [0.05, 6.07]		2.4 [0.98, 5.40]
17		2.9 [0.76, 8.90]		1.6 [0.51, 4.32]
18		1.9 [0.34, 7.52]		2.0 [0.74, 4.87]
19		0		0.8 [0.14, 3.17]
20		3.9 [1.25, 10.20]		1.6 [0.51, 4.32]
21		1.9 [0.34, 7.52]		1.2 [0.31, 3.76]
22		1.9 [0.34, 7.52]		1.2 [0.31, 3.76]
23		3.9 [1.25, 10.20]		0.4 [0.02, 2.56]

The proportion and 95% confidence interval is shown for participants in each subgroup illustrated in Figure 2 of the main text. Among the full sample of 353 participants, there were n(%) [CI] 60 (17.0) [13.3 – 21.4] participants with no observable command-following and +fMRI or +EEG, 43 (12.2) [9.1 – 16.2] participants with observable command-following and +fMRI or +EEG, 181, [51.3], (45.9 – 56.6) participants with no observable command-following and -fMRI and -EEG, and 69(19.6) [15.6 – 24.1] participants with observable command-following and -fMRI and -EEG.
Abbreviations: *CRS-R* Coma Recovery Scale-Revised, *EEG* electroencephalography, *fMRI* functional magnetic resonance imaging

Table S7: Cognitive Motor Dissociation Proportions by Site

Site	**All Participants Without Observable Command-Following** (Coma/vegetative state, minimally conscious state minus) **N=241**	**+fMRI or +EEG (cognitive motor dissociation)** **N (%) [95% CI]**
Weill Cornell Medicine, USA	22	10 (45%) [25.07 – 67.32]
Columbia University, USA	39	6 (15%) [6.41 – 31.20]
Massachusetts General Hospital, USA	15	5 (33%) [12.99 – 61.31]
Cambridge University Hospitals, UK	70	26 (37%) [26.13 – 49.57]
University and University Hospital of Liege, Belgium	52	12 (23%) [12.98 – 37.18]
Paris Brain Institute, France	43	1 (2%) [0.122 – 11.47]

Abbreviations: *EEG* electroencephalography, *fMRI* functional magnetic resonance imaging

Table S8: CRS-R Diagnosis, Assessment Method, Chronicity, and Etiology Stratified by fMRI and EEG Responses in Participants Without Observable Command-Following

	CRS-R Diagnosis			Cognitive Motor Dissociation Assessment Method			Chronicity		Etiology			
	All N=241	Coma, Vegetative State N=140	Minimally Conscious State Minus N=101	Assessed with fMRI N=140[b]	Assessed with EEG N=180[b]	Assessed with fMRI and EEG N=79[c]	<28 days post injury N=72	≥28 days post injury N=169	TBI N=108	Cardiac Arrest/anoxia N=45	Vascular Stroke/SAH N=48	Other N=40
+fMRI or +EEG (i.e., cognitive motor dissociation) N (%)[a]	60 (25%)	28 (20%)	32 (32%)	37 (26%)	28 (16%)	36 (46%)	12 (17%)	48 (28%)	39 (36%)	4 (9%)	9 (19%)	8 (20%)
-fMRI and -EEG N (%)	181 (75%)	112 (80%)	69 (68%)	103 (74%)	152 (84%)	43 (54%)	60 (83%)	121 (72%)	69 (64%)	41 (91%)	39 (81%)	32 (80%)

[a] all proportions are calculated from the number of participants indicated in the column heading. For example, of 241 participants without observable command-following (a CRS-R diagnosis of coma/vegetative state [unconscious] or minimally conscious state minus [minimally conscious state without command-following]), 60 (25%) had cognitive motor dissociation. "+fMRI or +EEG" indicates that at least one assessment (either fMRI or EEG regardless of whether participants had one or both of these assessments) was positive. "-fMRI and -EEG" indicates that for participants with fMRI only, the fMRI assessment was negative; for participants with EEG only, the EEG assessment was negative; for participants with both fMRI and EEG, both assessments were negative.

[b] values in the "Assessed with fMRI" column represent proportions of participants with +fMRI (top row) or -fMRI (bottom row) and values in the "Assessed with EEG" column represent proportions of participants with +EEG (top row) or -EEG (bottom row). Of N=140 with an fMRI assessment, N=79 also had an EEG assessment and of N=180 with an EEG assessment, N=79 also had an fMRI assessment, however, the results in the "Assessed with fMRI" column only report the fMRI results and the results in the "Assessed with EEG" column only report the EEG results.

[c] values in the "Assessed with fMRI and EEG" column represent proportions of the 79 participants who were assessed with both fMRI *and* EEG and were positive on fMRI, EEG, or both (top row) or were negative on both fMRI *and* EEG (bottom row)

Abbreviations: CRS-R C*oma Recovery Scale-R*evised; ICH *intracerebral hemorrhage*; IVH *intraventricular hemorrhage*; EEG *electroencephalography*; fMRI *functional magnetic resonance imaging*; SAH *subarachnoid hemorrhage*; TBI *traumatic brain injury*

Table S9: CRS-R Diagnosis, Assessment Method, Chronicity, and Etiology Stratified by fMRI and EEG Responses in Participants With Observable Command-Following

	CRS-R Diagnosis			Assessment Method			Chronicity		Etiology			
	All N=112	Minimally Conscious State Plus N=77	Emerged from Minimally Conscious State N=35	Assessed with fMRI N=75[b]	Assessed with EEG N=80[b]	Assessed with fMRI and EEG[c] N=43	<28 days post injury N=18	≥28 days post injury N=94	TBI N=68	Cardiac Arrest/anoxia N=12	Vascular Stroke/ SAH N=17	Other N=15
**+fMRI or +EEG N (%)[a]**	43 (38%)	26 (34%)	17 (49%)	26 (35%)	33 (41%)	25 (58%)	10 (56%)	33 (35%)	30 (44%)	1 (8%)	9 (53%)	3 (20%)
**-fMRI and -EEG N (%)**	69 (62%)	51 (66%)	18 (51%)	49 (65%)	57 (71%)	18 (42%)	8 (44%)	61 (65%)	38 (56%)	11 (92%)	8 (47%)	12 (80%)

[a] all proportions are calculated from the number of participants indicated in the column heading. For example, of 112 patients with observable command-following (a CRS-R diagnosis of minimally conscious state plus [minimally conscious state with command-following or intelligible speech] or emerged from minimally conscious state), 44 (38%) demonstrated covert command-following on fMRI, EEG, or both assessments. "+fMRI or +EEG" indicates that at least one assessment (either fMRI or EEG regardless of whether participants had one or both of these assessments) was positive. "-fMRI and -EEG" indicates that for participants with fMRI only, the fMRI assessment was negative; for participants with EEG only, the EEG assessment was negative; for participants with both fMRI and EEG, both assessments were negative.
[b] values in the "Assessed with fMRI" column represent proportions of participants with +fMRI (top row) or -fMRI (bottom row) and values in the "Assessed with EEG" column represent proportions of participants with +EEG (top row) or -EEG (bottom row). Of N=75 with an fMRI assessment, N=43 also had an EEG assessment and of N=80 with an EEG assessment, N=43 also had an fMRI assessment, however, the results in the "Assessed with fMRI" column only report the fMRI results and the results in the "Assessed with EEG" column only report the EEG results.
[c] values in the "Assessed with fMRI and EEG" column represent proportions of the 43 participants who were assessed with both fMRI *and* EEG and were positive on either fMRI, EEG, or both (top row) or were negative on both fMRI and EEG (bottom row)

Abbreviations: CRS-R C*oma Recovery Scale*-Revised; ICH *intracerebral hemorrhage*; IVH *intraventricular hemorrhage*; EEG *electroencephalography*; fMRI *functional magnetic resonance imaging*; SAH *subarachnoid hemorrhage*; TBI *traumatic brain injury*

Table S10: Concordance Between Command-Following on the CRS-R, fMRI, and EEG

**a. Agreement between evidence of observable command-following on the CRS-R and fMRI *or* EEG**		
N=353	fMRI and EEG Command-following NO (-fMRI and -EEG)	fMRI or EEG Command-following YES (+fMRI or +EEG)
Observable Command-Following on CRS-R – NO	181	60
Observable Command-Following on CRS-R –YES	69	43

**b. Agreement between evidence of observable command-following on the CRS-R and fMRI**		
N=215	fMRI command-following NO (-fMRI)	fMRI command-following YES (+fMRI)
Observable Command-Following on CRS-R – NO	103	37
Observable Command-Following on CRS-R–YES	49	26

**c. Agreement between evidence of observable command-following on the CRS-R and EEG**		
N=260	EEG Command-following NO (-EEG)	EEG Command-following YES (+EEG)
Observable Command-Following on CRS-R – NO	152	28
Observable Command-Following on CRS-R –YES	57	23

Agreement between evidence of command-following on the CRS-R, fMRI, and EEG was low. Kappa coefficient (95% confidence interval) was 0.14 (0.03, 0.25), 0.09 (-0.05, 0.22), and 0.15 (.02, 0.27), for tables a, b, and c, respectively. "+fMRI or +EEG" indicates that at least one assessment (either fMRI or EEG regardless of whether participants had one or both of these assessments) was positive. "-fMRI and -EEG" indicates that all assessments (fMRI, EEG, or both fMRI and EEG for participants who had both assessments) were negative. "Observable command-following on CRS-R NO" indicates a CRS-R diagnosis of coma/vegetative state (unconscious) or minimally conscious state minus (i.e., minimally conscious state without command-following). "Observable command-following on CRS-R YES" indicates a CRS-R diagnosis of minimally conscious state plus (minimally conscious state with command-following) or emerged from minimally conscious state. Outlined boxes indicate cognitive motor dissociation. Concordance is shaded in green; discordance is shaded in orange.

Abbreviations: CRS-R *Coma Recovery Scale*-Revised; EEG *electroencephalography*; fMRI *functional magnetic resonance imaging*

Table S11: Concordance Between Covert Command-Following on fMRI and EEG

**a. All participants with at least one fMRI and one EEG assessment**		
N=122	EEG Command-following NO (-EEG)	EEG Command-following YES (+EEG)
fMRI Command-following NO (-fMRI)	61	19
fMRI Command-following YES (+fMRI)	31	11

**b. Participants without observable command-following and at least one fMRI and one EEG assessment**		
N=79	EEG Command-following NO (-EEG)	EEG Command-following YES (+EEG)
fMRI Command-following NO (-fMRI)	43	10
fMRI Command-following YES (+fMRI)	21	5

**c. Participants with observable command-following and at least one fMRI and one EEG assessment**		
N=43	EEG Command-following NO (-EEG)	EEG Command-following YES (+EEG)
fMRI Command-following NO (-fMRI)	18	9
fMRI Command-following YES (+fMRI)	10	6

Agreement between evidence of command-following of fMRI and EEG was low across the entire sample and in the subgroup without observable command-following (coma, vegetative state, minimally conscious state without command-following) and the subgroup with observable command-following (minimally conscious state with command-following and emerged from minimally conscious state). Kappa coefficient (95% confidence interval) was 0.03 (-0.15, 0.20), 0.02 (-0.20, 0.21) and 0.04 (-0.26, 0.34) for tables a, b, and c, respectively. “+fMRI” and “+EEG” indicate a response to task-based fMRI or EEG, respectively, was detected. “-fMRI” and “-EEG” indicate a response to task-based fMRI or EEG, respectively, was not detected. “Without observable command-following” indicates a CRS-R diagnosis of coma/vegetative state (unconscious) or minimally conscious state minus (minimally conscious state without command-following). “With observable command-following” indicates a CRS-R diagnosis of minimally conscious state plus (minimally conscious state with command-following) or emerged from minimally conscious state. Concordance is shaded in green; discordance is shaded in orange.

Abbreviations: CRS-R *Coma Recovery Scale*-Revised; EEG *electroencephalography*; fMRI *functional magnetic resonance imaging*

SUPPLEMENTARY REFERENCES

1. Edlow BL, Chatelle C, Spencer CA, et al. Early detection of consciousness in patients with acute severe traumatic brain injury. Brain 2017;140:2399-414.
2. Egbebike J, Shen Q, Doyle K, et al. Cognitive-motor dissociation and time to functional recovery in patients with acute brain injury in the USA: a prospective observational cohort study. Lancet Neurol 2022;21:704-13.
3. Goldfine AM, Victor JD, Conte MM, Bardin JC, Schiff ND. Determination of awareness in patients with severe brain injury using EEG power spectral analysis. Clin Neurophysiol 2011;122:2157-68.
4. Bardin JC, Fins JJ, Katz DI, et al. Dissociations between behavioural and functional magnetic resonance imaging-based evaluations of cognitive function after brain injury. Brain 2011;134:769-82.
5. Forgacs PB, Conte MM, Fridman EA, Voss HU, Victor JD, Schiff ND. Preservation of electroencephalographic organization in patients with impaired consciousness and imaging-based evidence of command-following. Ann Neurol 2014;76:869-79.
6. Curley WH, Forgacs PB, Voss HU, Conte MM, Schiff ND. Characterization of EEG signals revealing covert cognition in the injured brain. Brain 2018;141:1404-21.
7. Bodien YG, Giacino JT, Edlow BL. Functional MRI motor imagery tasks to detect command following in traumatic disorders of consciousness. Front Neurol 2017;8:688.
8. Monti MM, Vanhaudenhuyse A, Coleman MR, et al. Willful modulation of brain activity in disorders of consciousness. N Engl J Med 2010;362:579-89.
9. Franzova E, Shen Q, Doyle K, et al. Injury patterns associated with cognitive motor dissociation. Brain 2023.
10. Jain P, Conte MM, Voss HU, Victor JD, Schiff ND. Low-level language processing in brain-injured patients. Brain Commun 2023;5:fcad094.
11. Cruse D, Chennu S, Chatelle C, et al. Bedside detection of awareness in the vegetative state: a cohort study. Lancet 2011;378:2088-94.
12. Cruse D, Chennu S, Chatelle C, et al. Relationship between etiology and covert cognition in the minimally conscious state. Neurology 2012;78:816-22.
13. Luppi AI, Craig MM, Coppola P, et al. Preserved fractal character of structural brain networks is associated with covert consciousness after severe brain injury. Neuroimage Clin 2021;30:102682.
14. Stender J, Gosseries O, Bruno M-A, et al. Diagnostic precision of PET imaging and functional MRI in disorders of consciousness: a clinical validation study. The Lancet 2014;384:514-22.
15. Goldfine AM, Victor JD, Conte MM, Bardin JC, Schiff ND. Bedside detection of awareness in the vegetative state. Lancet 2012;379:1701-2.
16. Cunningham C, Chen WC, Shorten A, et al. Impaired consciousness in partial seizures is bimodally distributed. Neurology 2014;82:1736-44.
17. Claassen J, Doyle K, Matory A, et al. Detection of brain activation in unresponsive patients with acute brain injury. N Engl J Med 2019;380:2497-505.
18. Lule D, Noirhomme Q, Kleih SC, et al. Probing command following in patients with disorders of consciousness using a brain-computer interface. Clin Neurophysiol 2013;124:101-6.
19. Lesenfants D, Habbal D, Chatelle C, Soddu A, Laureys S, Noirhomme Q. Toward an Attention-Based Diagnostic Tool for Patients With Locked-in Syndrome. Clin EEG Neurosci 2018;49:122-35.
20. Kondziella D, Friberg CK, Frokjaer VG, Fabricius M, Møller K. Preserved consciousness in vegetative and minimal conscious states: systematic review and meta-analysis. Journal of Neurology, Neurosurgery & Psychiatry 2016;87:485-92.

21. Tang Q, Lei J, Gao G, Feng J, Mao Q, Jiang J. Prevalence of persistent vegetative state in patients with severe traumatic brain injury and its trend during the past four decades: A meta-analysis. NeuroRehabilitation 2017;40:23-31.
22. Kondziella D, Amiri M, Othman MH, et al. Incidence and prevalence of coma in the UK and the USA. Brain Commun 2022;4:fcac188.
23. Løvstad M, Andelic N, Knoph R, et al. Rate of disorders of consciousness in a prospective population-based study of adults with traumatic brain injury. J Head Trauma Rehabil 2014;29:E31-43.
24. Turner-Stokes L, Rose H, Knight A, Williams H, Siegert RJ, Ashford SA. Prolonged disorders of consciousness: identification using the UK FIM + FAM and cohort analysis of outcomes from a UK national clinical database. Disabil Rehabil 2023;45:620-9.
25. Overbeek BUH, van Erp WS, Eilander HJ, Koopmans R, Lavrijsen JCM. Prevalence of the minimally conscious state among institutionalized patients in the Netherlands: A nationwide study. Neurology 2023;101:e2005-e13.
26. Irzan H, Pozzi M, Chikhladze N, et al. Emerging treatments for disorders of consciousness in paediatric age. Brain Sci 2022;12.
27. Molteni E, Canas LDS, Briand MM, et al. Scoping review on the diagnosis, prognosis, and treatment of pediatric disorders of consciousness. Neurology 2023;101:e581-e93.
28. Bellon PA, Bosso MJ, Echegaray JEC, et al. tracheostomy decannulation and disorders of consciousness evolution. Respir Care 2022;67:209-15.
29. Puggina AC, da Silva MJ. Patients with disorders of consciousness: vital, facial and muscular responses to music or messages. Rev Bras Enferm 2015;68:94-102, -10.
30. Chen WG, Li R, Zhang Y, et al. Recovery from prolonged disorders of consciousness: A dual-center prospective cohort study in China. World J Clin Cases 2020;8:2520-9.
31. Pan J, Xie Q, Qin P, et al. Prognosis for patients with cognitive motor dissociation identified by brain-computer interface. Brain 2020;143:1177-89.
32. Giacino JT, Katz DI, Schiff ND, et al. Practice guideline update recommendations summary: Disorders of consciousness. Neurology 2018;91:450.
33. van Erp WS, Lavrijsen JC, van de Laar FA, Vos PE, Laureys S, Koopmans RT. The vegetative state/unresponsive wakefulness syndrome: a systematic review of prevalence studies. European Journal of Neurology 2014;21:1361-8.
